# Competency-based evaluation tools for integrative medicine training in family medicine residency: a pilot study

**DOI:** 10.1186/1472-6920-7-7

**Published:** 2007-04-18

**Authors:** Benjamin Kligler, Mary Koithan, Victoria Maizes, Meg Hayes, Craig Schneider, Patricia Lebensohn, Susan Hadley

**Affiliations:** 1 Albert Einstein College of Medicine, Bronx, NY/University of Arizona School of Medicine Program in Integrative Medicine, Tucson, AZ, USA; 2 University of Arizona School of Medicine Program in Integrative Medicine, Tucson, AZ, USA; 3 University of Arizona School of Medicine Program in Integrative Medicine, Tucson, AZ, USA; 4 Oregon Health Sciences University, Portland, OR, USA; 5 Maine Medical Center, Portland, ME, USA; 6 University of Arizona School of Medicine Program in Integrative Medicine, Tucson, AZ, USA; 7 Middlesex Hospital/University of Connecticut, Middlesex, CT, USA

## Abstract

**Background:**

As more integrative medicine educational content is integrated into conventional family medicine teaching, the need for effective evaluation strategies grows. Through the Integrative Family Medicine program, a six site pilot program of a four year residency training model combining integrative medicine and family medicine training, we have developed and tested a set of competency-based evaluation tools to assess residents' skills in integrative medicine history-taking and treatment planning. This paper presents the results from the implementation of direct observation and treatment plan evaluation tools, as well as the results of two Objective Structured Clinical Examinations (OSCEs) developed for the program.

**Methods:**

The direct observation (DO) and treatment plan (TP) evaluation tools developed for the IFM program were implemented by faculty at each of the six sites during the PGY-4 year (n = 11 on DO and n = 8 on TP). The OSCE I was implemented first in 2005 (n = 6), revised and then implemented with a second class of IFM participants in 2006 (n = 7). OSCE II was implemented in fall 2005 with only one class of IFM participants (n = 6).

Data from the initial implementation of these tools are described using descriptive statistics.

**Results:**

Results from the implementation of these tools at the IFM sites suggest that we need more emphasis in our curriculum on incorporating spirituality into history-taking and treatment planning, and more training for IFM residents on effective assessment of readiness for change and strategies for delivering integrative medicine treatment recommendations. Focusing our OSCE assessment more narrowly on integrative medicine history-taking skills was much more effective in delineating strengths and weaknesses in our residents' performance than using the OSCE for both integrative and more basic communication competencies.

**Conclusion:**

As these tools are refined further they will be of value both in improving our teaching in the IFM program and as competency-based evaluation resources for the expanding number of family medicine residency programs incorporating integrative medicine into their curriculum. The next stages of work on these instruments will involve establishing inter-rater reliability and defining more clearly the specific behaviors which we believe establish competency in the integrative medicine skills defined for the program.

## Background

In 2000, a set of suggested curriculum guidelines [1] for family medicine residents in Complementary/Alternative Medicine (CAM) were published. These guidelines were endorsed by the Board of the Society of Teachers of Family Medicine in 1999, and represented the first published set of recommendations for residency-level teaching in this area. However, no well-defined set of measurable competencies has been identified and described for family medicine residents, and no competency-based evaluation tools have been developed and widely adopted to date. This article describes the ongoing efforts at six residency programs participating in a pilot program in "Integrative Family Medicine" to develop competencies in this area and the tools to measure them effectively. The term CAM has been widely replaced by "Integrative Medicine" (IM), which is defined as "healing-oriented medicine that takes account of the whole person (body, mind, and spirit), including all aspects of lifestyle. It emphasizes the therapeutic relationship and makes use of all appropriate therapies, both conventional and alternative. [2]"

Beginning in 2003, six residency programs joined with the University of Arizona Program in Integrative Medicine to collaboratively develop a four-year combined family medicine/integrative medicine residency program. The two goals of the program are to develop and implement an accredited model for a four year Integrative Family Medicine (IFM) training program which combines training in Integrative Medicine with conventional Family Medicine residency training, and to train physicians capable of practicing healing oriented medicine within the context of the whole person (mind, body, and spirit) and with the ability to employ all appropriate therapeutic options, both conventional and alternative.

The six sites participating in the IFM Program are the University of Arizona, Beth Israel/Albert Einstein College of Medicine, Maine Medical Center, Middlesex Hospital (University of Connecticut), Oregon Health Sciences University, and the University of Wisconsin. The Accreditation Council of Graduate Medical Education Family Medicine Residency Review Committee (RRC) awarded this pilot program experimental status in 2003. IFM residents from participating sites are identified in the early part of their PGY-2 year, and begin the integrative medicine component of the training experience in January of that year. Each of the six sites has a faculty member trained in integrative medicine who serves as the mentor for the resident's clinical experience during the program.

The core elements of the IFM program are outlined in Table 1. The details of the IFM curriculum have been described elsewhere, as has its early impact on recruitment at the six pilot sites [3]. This paper describes our efforts to develop and test a set of measurement tools to assess competencies for integrative medicine at the residency level.

**Table 1:** Core IFM Program Elements

• Distributed learning curriculum through participation in the Program in Integrative Medicine Associate Fellowship at the University of Arizona
∘ 1000 hours of distributed learning experiences (Internet-based activities, articles, textbooks, audio, botanical labs, and community experiences.)
∘ Three weeks of residential learning in Arizona distributed through PGY-2–4 years
• Integrative Medicine patient care continuity experience
∘ Continue primary care continuity clinic throughout all four years
∘ Participation in Integrative Medicine 'consultation' clinical experience in PGY 4 year
• Regularly scheduled Interdisciplinary Case Conference
• Involvement of key faculty who are trained in Integrative Medicine and embody the philosophy of practice of Integrative Medicine
• Emphasis on experiential learning including experiencing treatment modalities
• Achievement of competency in defined core curricular areas and proficiency or certification in at least one CAM modality
• A commitment to Self-Care demonstrated by each trainee developing a self-care wellness plan and reviewing it regularly with a faculty member

### Development of the IFM competencies

Several sets of competencies in integrative medicine, developed for use in other settings, were reviewed as we began to describe the required competencies for the IFM program. [4] In an iterative process of dialogue involving the faculty members at the six sites, we arrived at the set of competencies listed in Table 2.

**Table 2:** IFM Competencies with ACGME domains

**ACGME Core Competency**	**1**	**2**	**3**	**4**	**5**	**6**	**7**	**8**	**9**	**10**	**11**	**12**	**13**	**14**	**15**
Patient Care		x	x		x				x	x	x	x	x	x	
Medical Knowledge								x							
Practice-Based Learning & Improvement	x							x							x
Interpersonal & Communication Skills				x			x								
Professionalism	x	x													x
System-Based Practice						x	x								x

IFM Competencies

1. Practices self-care

2. Demonstrates self-awareness

3. Uses patient-centered care techniques

4. Uses communication skills that enhance the physician/patient relationship

5. Facilitates lifestyle changes in patients

6. Knows how to refer appropriately to CAM practitioners

7. Practices constructively and collaboratively with other health team member

8. Assesses scientific and historical evidence for allopathic as well as CAM approaches to specific diseases and syndromes

9. Integrates mind-body recommendations into practice appropriately

10. Integrates botanical recommendations into practice appropriately

11. Integrates nutrition recommendations into practice appropriately

12. Integrates physical activity recommendations into practice appropriately

13. Counsels and supports patients regarding spirituality

14. Composes and administers individualized integrative medicine treatment plans

15. Positively impacts their organization and/or environment (local, regional and/or national) with regard to Integrative Medicine

The most significant challenge to reaching consensus was deciding whether a number of the communication and patient-centered care competencies were already being adequately taught and measured in standard Family Medicine residency training, and thus should be omitted from the specific competencies for this program; or whether these areas – so central to the vision of integrative medicine – should be included here, despite the obvious overlap with the conventional family medicine curriculum. A decision was made to include both the more general communication competencies and the integrative medicine-specific competencies for this first round of evaluation. These competencies were then mapped to the six Accreditation Council for Graduate Medical Education competency domains: patient care, medical knowledge, practice based learning and improvement, interpersonal and communication skills, professionalism, and system-based practice. The IFM competencies and their relationship to the ACGME competencies are depicted in Table 2.

### Competency-based evaluation strategies

Once competencies were identified, evaluation strategies specific to the IFM program were developed based on faculty experience, a review of the evaluation literature, recommendations from the ACGME Outcomes Project,[5] and adoption of methods currently used in the family medicine residency programs of the participating institutions. These strategies include direct observation, reviews of written treatment plans, Objective Structured Clinical Examinations (OSCEs) to be done during the second and third residential weeks at the University of Arizona during the PGY 3 and PGY 4 years, and ongoing formative evaluation meetings with faculty. Knowledge-based evaluation tools used in the University of Arizona Integrative Medicine Fellowship curriculum were also incorporated. The overall evaluation plan is presented in Table 3, and each of the evaluation strategies developed for the IFM program is described.

**Table 3:** ACGME-linked IFM Competencies and evaluation strategies

**ACGME General Competencies**	**IFM General Competencies**	**Methods for Assessing Competence**
		I. Direct Observation
		II. Review Treatment Plan
		III. UA Integrative Medicine
		Fellowship Evaluation Methods
		IV. Faculty formative evaluations
		V. Standardized Patients (OSCE)
Patient Care	2, 3, 5, 9, 10, 11, 12, 13, 14	2: I, II, IV, V
		3: I, V
		5: I, II, V
		9: I, II, V
		10: I, II, V
		11: I, II, V
		12: I, II, V
		13: I, II, V
		14: I, II, III, V
Medical Knowledge	8	8: II, III, IV, V
Practice-Based Learning & Improvement	1, 8, 15	1: III, IV
		8: II, III, IV, V
		15: IV
Interpersonal and Communication Skills	4, 7	4: I, V
		7: I, IV
Professionalism	1, 2, 15	1: III, V
		2: I, II, IV, V
		15: IV
System-Based Practice	6, 7, 15	6: I, II, V
		7: I, IV
		15: IV

#### Direct observation evaluation tool

We developed a direct observation checklist that delineates the specific behaviors expected of IFM participants in the direct care of patients. Behaviors included in that instrument are reflective of program competencies and move across the ACGME General Competencies. The tool builds on a number of other previous efforts in assessing competence in graduate medical education, including the widely-used Mini-CEX. [6] The tool developed here expands on these efforts by incorporating specific behaviors reflecting the competencies in integrative medicine outlined above. Evaluating faculty rate these behaviors as emerging (beginning to show this skill), established (basic knowledge/skills attained and demonstrated routinely) and integrated (uses knowledge/skills flexibly as part of an overall repertoire). If behaviors have not been observed directly by the faculty or if input is insufficient reflection of their competency in these areas, behaviors are rated as DNO (did not observe). Behaviors that are repeatedly rated as DNO will be deleted in the finalized version of the IFM Direct Observation tool.

Each resident is evaluated approximately semi-annually; scores for each resident are compared to expected outcomes based on year of residency and then compared across years of training to determine professional growth and change. To emphasize the formative aspect of the methodology, each direct observation experience is followed by a debriefing session, which incorporates both (a) review of the behaviors observed by faculty and (b) reflection about the encounter experience. We believe such reflection is critical to good practice of integrative medicine. In the Debrief component of the Direct Observation evaluation, the resident is asked to describe: (a) How did you feel during the encounter? (b) What, if anything, did this encounter teach you about yourself? and (c) Reflecting back, is there anything else you could have done to enhance healing during this encounter?

#### Treatment plan evaluation tool

Similarly, we developed a treatment plan evaluation tool that delineates the specific behaviors and language expected of IFM participants when developing and modifying evidence-based integrative treatment plans for specific patients. The same rating scale is used for the behaviors, which again are linked to the IFM program competencies as well as the ACGME General Competencies. Two treatment plans from each resident are evaluated annually. These scores are shared with the resident during a debriefing session.

#### Objective Structured Clinical Examinations (OSCEs)

The OSCE component of our evaluation strategy has been implemented via two OSCE experiences: the first, OSCE I, takes place in the middle of the PGY-3 year, and the second, OSCE II, in the middle of the PGY-4 year. OSCE I examines integrative medicine history-taking skills; OSCE II assesses the development of an integrative medicine treatment plan and the effective communication of that plan to the patient. For OSCE I, residents are presented with a patient experiencing migraines; for OSCE II, with a patient at risk for cardiovascular disease. Each OSCE represents an encounter with a single Standardized Patient. We do recognize that using a single patient raises potential problems with content specificity and with attaining a generalizable estimate of clinical performance; however for this initial round of OSCE implementation logistical constraints limited us to a single patient encounter. We hope to develop multiple OSCE stations for future iterations of this process.

The data from the first implementation of OSCE I (2005) were reported elsewhere [4], and led to a significant revision of OSCE I (2006) for the next class, which is presented here. Additionally, the first implementation of OSCE II (Fall 2005) is reported here. OSCE behaviors for both OSCE II (2005) and OSCE I (2006) were again linked to IFM program competencies and ACGME General Competencies.

## Methods

The direct observation (DO) and treatment plan (TP) evaluation tools were implemented by faculty at each of the six sites during the PGY-4 year (n = 11 on DO and n = 8 on TP). The OSCE I was implemented first in 2005 (n = 6), revised and then implemented with a second class of IFM participants in 2006 (n = 7). OSCE II was implemented in fall 2005 with only one class of IFM participants (n = 6). This pilot study was approved by the University of Arizona Institutional Review Board.

Data from the initial implementation of these tools were then examined (a) to attempt to evaluate the effectiveness of our teaching and (b) to begin to evaluate the actual tool for its utility in our evaluation process. If residents performed poorly on any competencies across IFM sites and evaluation strategies, this might represent a deficiency in our teaching program. Contrastingly, if particular behaviors could not be evaluated consistently across sites (a given competency was repeatedly rated as DNO), this might mean that the competency (a) was not clearly defined or stated or (b) that the competency is not one well-suited to the type of evaluation (direct observation, review of treatment plans or standardized patients) for which it was proposed. Each of these findings would be used to suggest changes to the next iteration of this evaluation tool. Descriptive statistics were used to describe the data collected from the DO and TP tools.

## Results

### Direct observation and treatment plan evaluation tools

In order to evaluate the effectiveness of teaching and to determine areas of weakness within the training program, scores for individual competencies on the Direct Observation and Treatment Plan Evaluation Tools across participants/program sites were examined. We hypothesized that such weakness would emerge as consistently low scores across sites for a given item; for the purposes of this analysis, we considered items where the mean score was 2.5 (83%) or below out of a possible three. We then examined the data across sites for behaviors that observers consistently (>= 50% of the time) identified as "unable to evaluate" or "did not observe" in order to evaluate the utility of the evaluation tools. The results of the Direct Observation evaluation are listed in Table 4, and the results of the Treatment Plan evaluation are reported in Table 5.

**Table 4:** Scores on Direct Observation Evaluation Tool Across Program Sites (n = 11)

**Competency and Behaviors**	**Mean Score (range 1–3)**	**Standard Deviation**	Percent Rated as DNO*
**Direct Observation Evaluation Tool**			

***Uses patient-centered care techniques (Competency #3)***			

a. Takes a history which includes the patient's understanding of their illness	2.5**	0.50	0%
b. Incorporates information about family, community, and occupational factors into history-taking	2.9	0.30	0%
c. Inquires regarding role of stress in patient's health and self-care/stress management strategies	2.9	0.32	0%
d. Inquires about sources of pleasure or happiness in patient's life	2.7	0.47	0%
e. Inquires about care from other practitioners or healers and the impact of this on the patient	2.6	0.50	0%
f. Integrates prevention and health promotion into the visit	2.7	0.50	33.3%

***Uses communication skills that enhance the physician/patient relationship (Competency #4)***			

a. Uses appropriate introduction	2.9	0.30	0%
b. Uses appropriate eye contact, body language, physical distance/proximity	2.9	0.30	0%
c. Appropriately uses non-medical terms and clear language	2.8	0.42	0%
d. Demonstrates empathy and compassion	2.8	0.42	0%
e. Uses open-ended questions, allowing adequate time for patient's responses	2.9	0.30	0%

***Facilitates lifestyle changes in patients (Competency #5)***			

a. Assesses readiness for change	2.6	0.52	0%
b. Negotiates prioritization of areas in need of change	2.4**	0.79	12.5%
c. Identifies potential obstacles and mitigating strategies	2.7	0.44	2.5%
d. Formulates plan that is appropriate to the stage the patient is at	2.5**	0.79	22.2%

***Practices constructively and collaboratively with other health team member (Competency #7)***			

a. Communicates respectfully with other members of the team	2.6	0.58	62.5%
b. Actively solicits input on patient care from other team members	2.6	0.58	66.7%

***Integrates mind-body recommendations into practice appropriately (Competency #9)***			

a. Inquires about previous and current use and response to mind-body therapies	2.9	0.30	0%
b. Discusses potential applications of mind-body therapies to specific patient	2.8	0.42	9.1%
c. Identifies potential obstacles and mitigating strategies to the use of mind-body therapies	2.6	0.52	11.1%
d. Demonstrates/teaches mind-body technique(s) to patients or refers patient appropriately	2.6	0.55	37.5%

***Integrates botanical recommendations into practice appropriately (Competency #10)***			

a. Inquires about previous and current use and response to botanical therapies	2.9	0.32	0%
b. Discusses potential applications of botanical therapies specific to patient	2.5**	0.55	25.0%
c. Identifies potential obstacles and mitigating strategies to the use of botanicals	2.3**	0.58	57.1%
d. Recommends botanicals to patients or refers patients appropriately	2.3**	0.58	57.1%

***Integrates nutrition recommendations into practice appropriately (Competency #11)***			

a. Inquires about previous and current use and response to nutritional therapies	2.9	0.30	0%
b. Discusses potential applications of nutritional therapies to specific patient	2.8	0.38	22.2%
c. Identifies potential obstacles and mitigating strategies to the use of nutritional therapies	2.7	0.49	22.2%
d. Recommends nutritional interventions to patients or refers appropriately	3.0	0.00	33.3%

***Integrates physical activity recommendations into practice appropriately (Competency #12)***			

a. Inquires about previous and current use and response to physical activity	2.7	0.44	10.0%
b. Discusses potential applications of physical activity to specific patient	2.8	0.41	25.0%
c. Identifies potential obstacles and mitigating strategies to the use of physical activity	2.6	0.52	25.0%
d. Recommends physical activity interventions to patients or refers appropriately	2.7	0.50	50.0%

***Counsels and supports patient regarding spirituality (Competency #13)***			

a. Inquires about previous and current spiritual or religious practice in a sensitive manner if indicated	3.0	0.00	33.3%
b. Discusses potential relevance of spirituality to health if indicated	2.7	0.50	50.0%
c. Refers for pastoral care or other spiritual approach when appropriate	2.5**	0.71	75.0%

***Composes and administers individual integrative medicine treatment plan (Competency #14)***			

a. Produces a coherent and readable written treatment plan for new patients and reviews this plan with faculty	3.0	0.00	12.5%
b. Incorporates evidence-based approach into treatment plan, when possible	2.5**	0.55	25.0%
c. Builds in to treatment plan comments on what patient already is doing well and where they have already shown strength and resources	2.8	0.45	37.5%
d. Incorporates feedback from faculty effectively into treatment plan	3.0	0.00	22.2%
e. Communicates plan clearly to patient, including discussion of potential obstacles to and facilitators of its implementation	2.8	0.41	22.2%

* DNO = Did Not Observe

** Scores ≤ 2.5 were determined to be below acceptable

**Table 5:** Scores on Treatment Plan Evaluation Tool Across Program Sites (n = 9)

**Competency and Behaviors**	**Mean Score (range 1–3)**	**Standard Deviation**	Percent Rated as DNO*
**Direct Observation Evaluation Tool**			

***Facilitates lifestyle changes in patients (Competency #5)***			

a. Assesses motivational stage of patient	2.2**	0.46	0%
b. Uses appropriate language in recommending lifestyle changes to patients	2.5**	0.53	12.5%

***Knows how to refer appropriately to CAM practitioners (Competency #6)***			

a. Refers when condition is potentially amenable to therapy utilized by practitioner	2.7	0.46	0%
b. Refers for a therapy that is acceptable to patient based on history	2.8	0.38	12.5%
c. Refers to a practitioner who is licensed, where appropriate	2.7	0.76	12.5%
d. Provides explanation/preview of therapy	2.3**	0.74	0%
e. Provides plan for follow up/communication	2.8	0.55	28.5%

***Assesses scientific and historical evidence for allopathic as well as CAM approaches to specific diseases and syndromes (Competency#8)***			

a. Provides evidence from scientific studies where appropriate (i.e., citations demonstrating use of information technology)	1.8**	0.98	25.0%
b. Provides explanation of traditional uses where appropriate	2.3**	0.76	12.5%
c. Provides explanation of potential benefits and risks including adverse effects/interactions where appropriate	2.1**	0.75	25.0%

***Integrates mind-body recommendations into practice appropriately (Competency #9)***			

a. Determine applicability of mind-body medicine methods to individual patients	2.7	0.46	0%
b. Gives rationale for utilizing mind-body method(s)	2.6	0.74	0%

***Integrates botanical recommendations into practice appropriately (Competency #10)***			

a. Determine applicability of botanical medicine methods to individual patients	2.8	0.38	12.5%
b. Gives rationale for utilizing botanical(s)	2.8	0.38	12.5%

***Integrates nutrition recommendations into practice appropriately (Competency #11)***			

a. Determine applicability of nutritional intervention(s) to individual patients	2.9	0.35	0%
b. Gives rationale for utilizing nutritional intervention(s)	2.7	0.46	0%

***Integrates physical activity recommendations into practice appropriately (Competency # 12)***			

a. Determine applicability of physical activity recommendation(s) to individual patients	3.0	0.00	0%
b. Gives rationale for utilizing physical activity recommendation(s)	2.8	0.38	0%

***Counsels and support patients regarding spirituality (Competency #13)***			

a. Determine applicability of spiritual intervention(s) to individual patients	2.0**	0.82	42.9%
b. Gives rationale for utilizing spiritual intervention(s)	2.3**	0.58	57.1%

***Composes and administer individualized integrative medicine treatment plans (Competency # 14)***			

a. Prepares written management plan for patients (and colleagues)	2.9	0.35	0%
b. Addresses specific health problem and emphasizes health maintenance and disease prevention	2.8	0.41	14.2%
c. Utilizes appropriate patient-oriented language	3.0	0.00	0%
d. Reviews plan with patient to enhance understanding and negotiate next steps	3.0	0.00	25.0%
e. Demonstrates caring and respectful behaviors	3.0	0.00	25.0%
f. Plans appropriate monitoring and follow-up	3.0	0.00	25.0%

* DNO = Did Not Observe

** Scores ≤ 2.5 were determined to be below acceptable

### OSCE results

Results from the OSCE I (2005) were previously reported [4]. Results of OSCE I (2006) are displayed in Table 6. The range of scores on OSCE I (2005) was 80–100%, with a mean score of 90%. As can be seen from the sampling of scores in Table 6, the change in scores between 2005 and 2006 suggests that these revisions in OSCE competencies were helpful; competencies now identified in OSCE I are more specific to integrative medicine and less repetitive of skills associated with family medicine residency. In OSCE I (2005) there was almost no one who failed to meet the "too-basic" patient-care and communication competencies. Total scores in OSCE I (2006) dropped, providing much more useful information for us as to what areas of the IFM participants training need more attention in the PGY-2/3 years.

**Table 6:** OSCE I (2006) IFM Competency Scores*

**OSCE Behavior/Competency**	**Percent Participants Correctly Demonstrating Behaviors**
Inquires about past medical history	71%
Inquires about family history	43%
Inquires about current and past use of complementary/alternative health care	57%
Explores/inquires/comments about the possibility of co-existing depression	57%
Commented on interaction between food and migraines	57%
Inquires about how the migraines are affecting his/her performance at work	29%
Inquires about how the migraines are affecting his/her relationship with spouse	29%
Inquires about the relationship between neck pain and migraines	57%
Comments that stress may be related to the occurrence of headaches	57%
Asks if patient has tried massage	57%
Communicates in some fashion that the provider will work with you to make you feel better/make life better	71%

* The previously published scores on OSCE 1 (2005) are not included here since these reflected an entirely different OSCE case.

Outcomes from OSCE I (2006) also suggest that although we can assume basic communication skills as a prerequisite already established in our IFM participants by the middle of PGY-3 year, we still have significant work to do in the area of comprehensive history-taking, both conventional and integrative. Perhaps most surprising is that only 57% of participants asked about current and past use of CAM, only 57% adequately screened for depression, and only 57% recognized that both stress and neck pain could be contributing to the migraines in the OSCE patient. Only 29% inquired in depth on how the migraines might be affecting work or personal relationships for the patient.

OSCE II was designed to assess knowledge base and treatment planning and communication skills. OSCE II (2005) results (Table 7) suggest that IFM participants need more training regarding the role of spirituality in cardiovascular health, as well as in the use of fish oils, botanical medicines, and nutritional supplements for cardiovascular risk reduction. They also suggest – at least as it pertains to diet change and exercise recommendations – that despite our emphasis on patient empowerment and informed choice, residents may need to be reminded to include explanations of why a set of recommendations might be beneficial to the patient in their delivery of those recommendations. Of course it should be acknowledged that because of the small sample size, the fragility of these percentages in our OSCE results makes drawing definitive conclusions difficult.

**Table 7:** OSCE II (2005) IFM Competency Scores

**OSCE Behavior/Competency**	**Percent Participants Correctly Demonstrating Behaviors**
Discussed your [patient's] spirituality and its impact on adoption of recommendations	60%
Suggested at least one diet change (low glycemic index diet, ↑ fruit and vegetables, ↓ saturated fat, ↓ carbohydrates, ↓ alcohol	100%
Discussed why this diet change would be good for you	60%
Suggested use of omega-3 fatty acids/fish oil	40%
Suggested at least one botanical (garlic, policosanol, gugulipid)	60%
Suggested increased physical activity (aerobics, yoga, weight lifting)	100%
Discussed why exercise would be good for you	60%
Suggested use of at least one supplement	40%

## Discussion

Two fundamental goals directed our development of competency-based evaluation tools for family physicians training in integrative medicine. Our immediate goal was to develop an effective evaluation strategy for the participants in the Integrative Family Medicine pilot program to meet internal evaluation needs as well as those of the Family Medicine Residency Review Committee (RRC). Our long-term goal is to contribute measurement tools for broad use in Family Medicine Residency programs. As more integrative medicine educational content is integrated into conventional family medicine teaching, the need for effective evaluation strategies grows. Current curriculum being introduced into family medicine residency training includes the use of botanicals and nutritional supplements, the use of specific dietary strategies such as the anti-inflammatory diet and the use of mind-body approaches. Evidence-based discussion of these areas regularly appears in American Family Physician reviews, [7] and the number of questions on these topics on the American Board of Family Practice certification exam continues to increase. A recent Institute of Medicine report recommends that "...health profession schools (e.g. schools of medicine, nursing, pharmacy, and allied health) [should] incorporate sufficient information about CAM into the standard curriculum at the undergraduate, graduate and post graduate levels to enable licensed professionals to competently advise their patients about CAM."[8]

However there are no competency-based evaluation tools that effectively assess competencies in evidence-based integrative medicine for use in residency programs. Our intention is to share our experience in developing and utilizing tools that may be modified for use in residency programs.

We found the direct observation checklist a useful tool, particularly when incorporating a debriefing session following the observation. The addition of this element allowed us to re-emphasize the "personal process" dimension of the integrative interview which incorporates an emphasis on reflection and countertransference as clinical skills essential to the integrative approach. The limitation, as with all such tools, relates to the difficulty of standardizing across observers and sites the exact behaviors that must be observed to demonstrate competency in a certain area. We plan to establish better face validity for the next iteration of these tools by defining exact behaviors more clearly using input from experts in the field of integrative medicine outside of the IFM faculty. Whereas the six IFM programs all have an "expert" faculty member who can rely on their own experience in assessing competency in herbal prescribing, or incorporation of mind-body strategies into treatment planning, other residencies might lack such expertise among the faculty. Therefore defining more clearly the behaviors which represent competency will be critical to the potential broader use of this instrument in non-IFM residencies.

The reliability of these tools also needs to be established if they are to enter wider use in residency programs. At our next faculty meeting, we plan to hold an "observation rating session" in which the six faculty in the program all observe one resident in a patient encounter and rate their performance using the DO tool. We will then use this data to measure the inter-rater reliability for this tool. We are also planning a similar process to establish inter-rater reliability for the treatment plan evaluation tool during the coming year.

Our initial OSCE experience revealed that we were measuring basic patient-centered communication skills which more properly belong in the domain of conventional family medicine. IFM participants in the initial OSCE I (2005) confirmed that by the PGY-3 year these skills have been thoroughly mastered and should be considered prerequisites for the IFM training rather than objectives for the program. Our second iteration of the OSCE I (2006) focused more narrowly on integrative medicine history-taking – competencies 6 through 14 specifically – and was much more effective in delineating strengths and weaknesses in our residents' performance. We are hopeful that as we develop new OSCEs more focused on these specific integrative medicine competencies that these might be useful, either wholly or in part, for residencies to incorporate into their ongoing OSCE assessment strategies.

Recognizing the infancy of both the IFM program and these evaluation strategies – and the fact that we do not know if areas of weakness in performance identified are a function of actual resident performance or of a weakness in the design of our tools – there were two areas of relatively weak performance which appeared both on the DO/TP assessment and on the OSCEs. First, as discussed above, there is an apparent pattern of weakness in the area of how treatment recommendations are communicated to patients – i.e. how often the obstacles to implementation are discussed adequately, and how effective residents are in assessing readiness for change. Second is the fact that apparently spirituality is often not discussed with patients by IFM participants. Since both of these findings appear in both evaluation settings, our hypothesis is that these do represent real weaknesses in performance rather than weaknesses in our measurement strategies. Of course it is also possible that due to our small "n" and variability across sites, these results represent evaluation artifact or confounding rather than actual weakness in performance and/or curriculum. We plan to more actively address both of these areas in our curriculum in the coming year, and our hope is that this will lead to improvement in competency scoring on the next round of DO/TP and OSCE evaluation scheduled for 2007 and to a change in how our rising PGY-4s perform in these areas.

## Conclusion

The Integrative Family Medicine program has created a unique need and opportunity to develop specific integrative medicine competencies for residents as well as the tools to measure them. To date, OSCEs exploring the expanded integrative history and treatment plan have been developed, assessed and modified. These skills have been further assessed with a direct observation tool and a treatment plan review tool. As these tools are refined further they are likely to be of value to the expanding number of family medicine residency programs incorporating integrative medicine into their curriculum.

The next stages of work on these instruments will involve establishing inter-rater reliability by having faculty independently rate the same clinical encounter, and establishing face validity more clearly by defining more clearly, using opinion solicited from experts in the field of integrative medicine outside of the IFM faculty, the specific behaviors which we feel represent competency in the integrative medicine skills we have defined for the program.

## Competing interests

The author(s) declare that they have no competing interests.

## Authors' contributions

All of the authors participated in conceiving the evaluation process and tools described in the article. BK coordinated the writing of the manuscript and assisted in the analysis of the evaluation data. MK was responsible for analysis of the evaluation data and contributed to the writing of the methods, results and discussion sections. VM oversees the IFM program and contributed to the writing of the Background and Discussion sections. MH, CS, PL and SH were responsible for collecting the evaluation data on the participants and contributed significantly to the writing of the article. All authors read and approved of the final manuscript.

## Pre-publication history

The pre-publication history for this paper can be accessed here:


